# Supplementary material for: Assessing the Health Benefits of Physical Activity Due to Active Commuting in a French Energy Transition Scenario
Source: Int J Public Health. 2022 Jul 12;67:1605012. doi: 10.3389/ijph.2022.1605012 (PMC9314562; doi:10.3389/ijph.2022.1605012)
Supplement: Supplementary file 1 [file DataSheet3.docx]

**
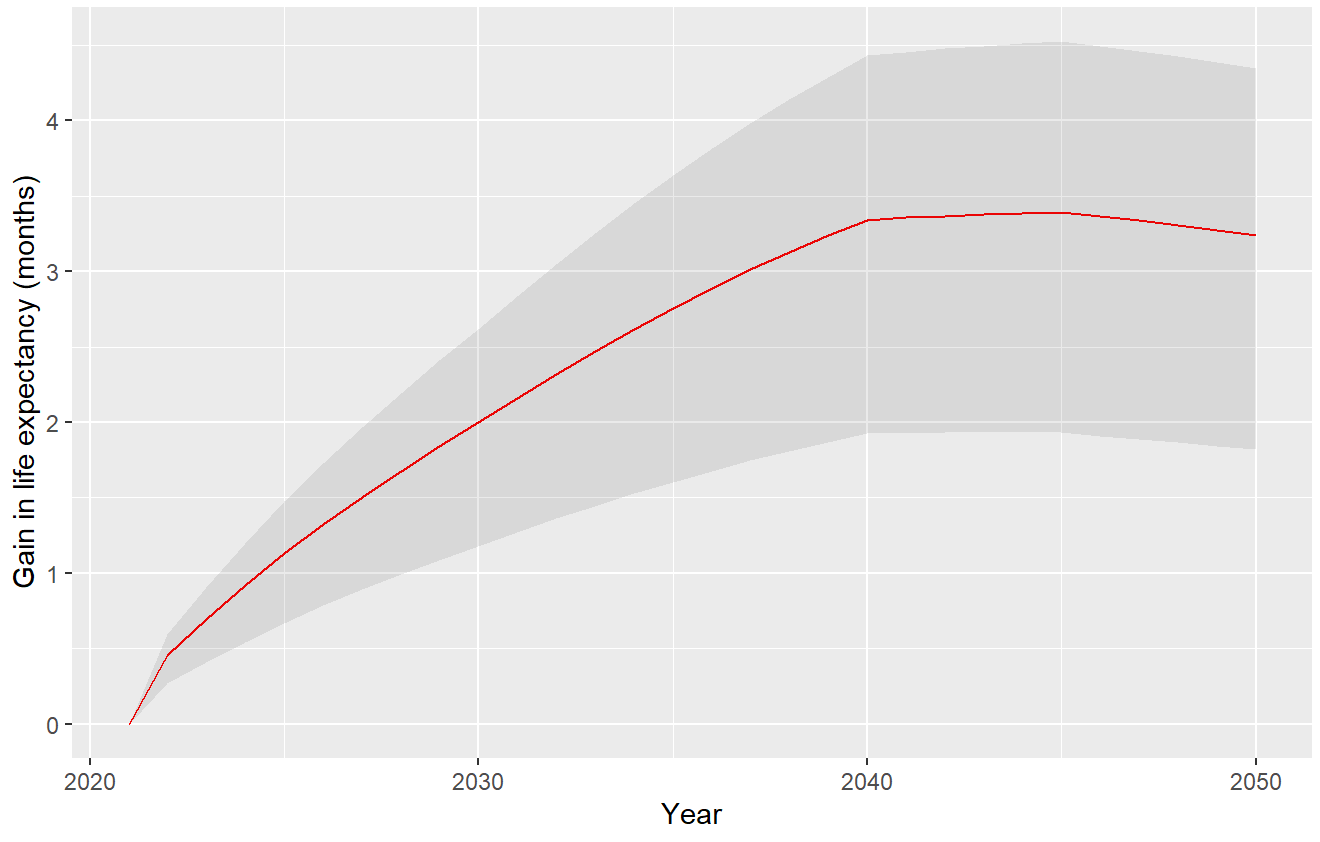
**

**Supplementary Figure 2 : Projected life expectancy gain, in months, expected by increased physical activity in the negaWatt scenario, 2020-2050 (grey: uncertainty interval).**
