# Supplementary material for: Assessing the Health Benefits of Physical Activity Due to Active Commuting in a French Energy Transition Scenario
Source: Int J Public Health. 2022 Jul 12;67:1605012. doi: 10.3389/ijph.2022.1605012 (PMC9314562; doi:10.3389/ijph.2022.1605012)
Supplement: Supplementary file 2 [file DataSheet2.docx]

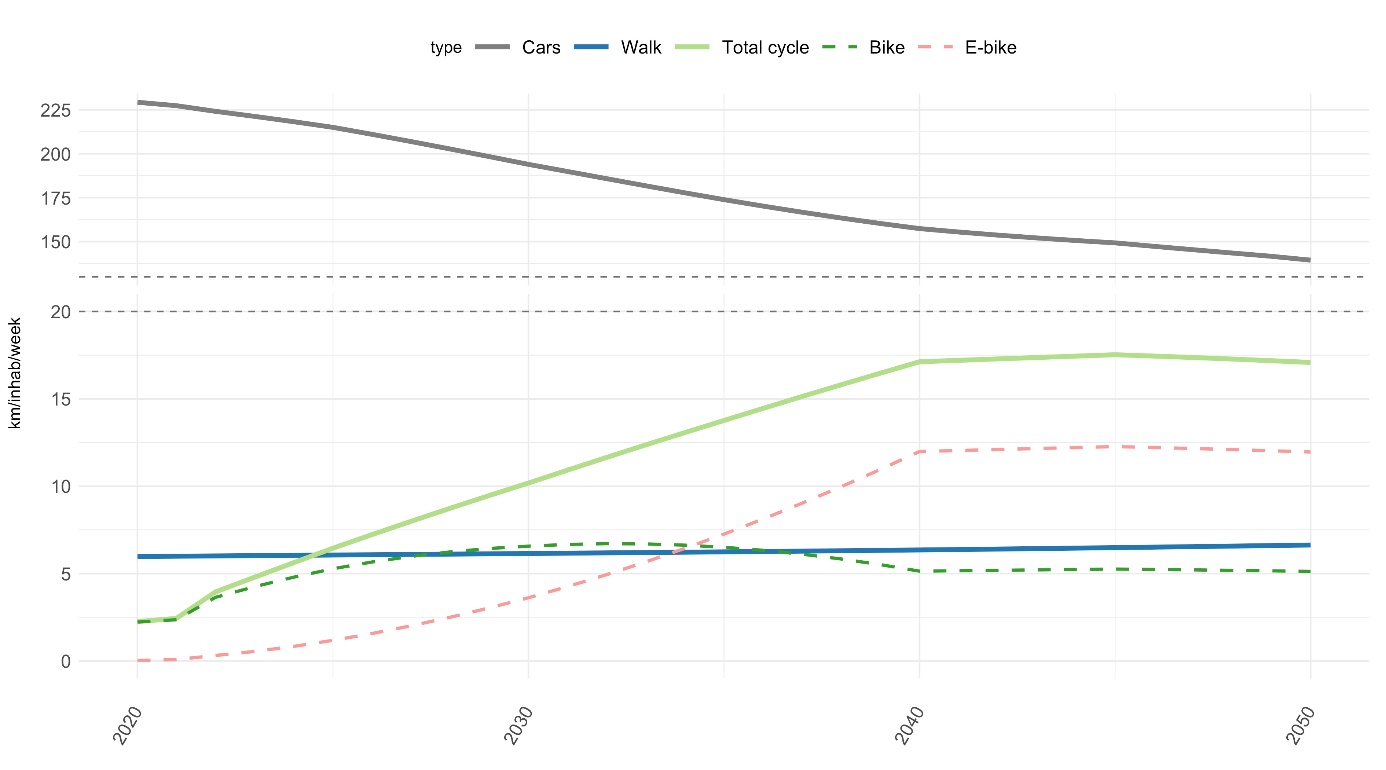


**Supplementary figure 1: Evolution of weekly mileage per transportation mode (negaWatt scenario, 2020-2050).**
