# Supplementary material for: Assessing the Health Benefits of Physical Activity Due to Active Commuting in a French Energy Transition Scenario
Source: Int J Public Health. 2022 Jul 12;67:1605012. doi: 10.3389/ijph.2022.1605012 (PMC9314562; doi:10.3389/ijph.2022.1605012)
Supplement: Supplementary file 3 [file DataSheet1.DOCX]

**Supplementary Text 1 : Distributing yearly projected mileage across ages and bike types**

*Distributing yearly projected mileage across ages*

In order to allocate the yearly mileages of walking and cycling (total km for classical and E-bike) projected in the negaWatt scenario across ages in a quite homogeneous, though realistic way, we used the shape of the age-distribution of active transportation from Denmark, one of the country with the highest cycling level in Europe. More specifically, we used the distribution of the relative contribution of each age group to the global mileage of walking and cycling as a target distribution.

We obtained the Danish distribution of the yearly number kilometres cycled per person per age from […]. Let us note ${km\_pp}_{a}^{Den}$ the yearly number of kilometres cycled per person, where $a$ indexes the age group.

Using the first age group as a reference, we calculated the vector $\rho$ that gives, for each age group, the ratio of the yearly number of kilometres cycled per person as compared to the first age group:

$$\forall a \in\left( 2,n \right), \rho_{a}^{Den}=\frac{{km\_pp}_{a}^{Den}}{{km\_pp}_{a}^{Den}}$$

In order to obtain a French distribution that matches the Danish one independently of the yearly mileage and independently of the population age structure, we want the ratio between similar age groups from both countries to be equal:

$$\forall a \in\left( 1,n \right), \rho_{a}^{Den}= \rho_{a}^{Fr}\leftrightarrow\frac{{km\_pp}_{a}^{Den}}{{km\_pp}_{1}^{Den}}=\frac{{km\_pp}_{a}^{Fr}}{{km\_pp}_{1}^{Fr}}$$

The energetic transition scenario used as input implies a yearly total number of kilometres cycled, and demographic projections are used to assume, for each year, a specific age structure. Therefore, we have to solve for each year the set of values of ${km\_pp}_{a}^{Fr}$ that ensures the match between the Danish and French age-specific cycling distribution, given a specific value of yearly total number of kilometres cycled ${KM}^{Fr}$ and a specific age structure. This age structure is represented by the set of values ${pop}_{a}^{Fr}$, the number of people in the age group i.

For each year, we thus have

$${KM}^{Fr}=\sum_{a} {pop}_{a}^{Fr}\times{km\_pp}_{a}^{Fr}$$

And $\forall a \in\left( 2,n \right), {km\_pp}_{a}^{Fr}= \rho_{a}\times$ ${km\_pp}_{1}^{Fr}$

Thus:

$${KM}^{Fr}={km\_pp}_{1}^{Fr}\times\left( {pop}_{1}^{Fr}+ \sum_{a=2}^{n} \rho_{a}\times{pop}_{a}^{Fr} \right)$$

And thus :

$${km\_pp}_{1}^{Fr}= \frac{{KM}^{Fr}}{{pop}_{1}^{Fr}+ \sum_{a=2}^{n} \rho_{a}\times{pop}_{a}^{Fr}}$$

We can now solve it for any other group year :

$$\forall k \in\left( 2,n \right), {km\_pp}_{k}^{Fr}= \frac{\rho_{k}\times{KM}^{Fr}}{{pop}_{1}^{Fr}+ \sum_{a=2}^{n} \rho_{a}\times{pop}_{a}^{Fr}}$$

The similar formulas were used to distribute the yearly global walking mileage.

*Distributing yearly projected cycling mileage between bike types across ages*

For each year, the negaWatt scenario projects a total number of kilometres cycled, ${KM}^{Fr}$, and an overall proportion of these that are cycled using E-bike, $\Pi$. This proportion is not supposed to be constant across ages as previous international studies documented a significant age difference between users of classical vs. E-bike, thereafter noted $\delta$. This difference has been documented (ref) the former being on average older by 6y. We thus estimated the vector $\pi_{a}$ representing the proportion of kilometre cycled using E-bike for each age $a$.

This vector has to fulfil two conditions. First, after distributing the overall total of kilometres cycled across ages as explained above, the set of values $\pi_{a}$ applied to age-specific cycle mileages is expected to yield to the overall value of $\Pi$ :

$$\frac{\sum_{i} {pop}_{i}\times{km\_pp}_{i}\times\pi_{i}}{{KM}^{Fr}}=\Pi$$

Second, the age difference of the users of each bike type weighted by the age-specific mileage cycled had to equal :

$\frac{\sum_{a} a\times{km\_pp}_{a}\times\pi_{a}}{\sum_{a} {km\_pp}_{a}\times\pi_{a}}$ **-** $\frac{\sum_{a} a\times{km\_pp}_{a}\times{(1-\pi}_{a})}{\sum_{a} {km\_pp}_{a}\times(1-\pi_{a})}\boldsymbol{=}\delta$

We assumed that the set of parameters $\pi_{a}$ was a linearly defined by age :

$$\pi_{a}=x\times a+y$$

We used a numerical optimization algorithm (the R *optim*() function) to find the parameters a and b fulfilling these conditions.
